# Comparative Genomic Analysis and Species Delimitation: A Case for Two Species in the Zoonotic Cestode *Dipylidium caninum*

**DOI:** 10.3390/pathogens12050675

**Published:** 2023-05-03

**Authors:** Jeba R. J. Jesudoss Chelladurai, Aloysius Abraham, Theresa A. Quintana, Deb Ritchie, Vicki Smith

**Affiliations:** 1 Department of Diagnostic Medicine/Pathobiology, College of Veterinary Medicine, Kansas State University, Manhattan, KS 66506, USA; 2 Department of Biotechnology, Alagappa University, Karaikudi 630003, India

**Keywords:** cestode, *Dipylidium caninum*, cat and dog, genome comparison, species delimitation, flea tapeworm

## Abstract

*Dipylidium caninum* (Linnaeus, 1758) is a common zoonotic cestode of dogs and cats worldwide. Previous studies have demonstrated the existence of largely host-associated canine and feline genotypes based on infection studies, differences at the 28S rDNA gene, and complete mitochondrial genomes. There have been no comparative genome-wide studies. Here, we sequenced the genomes of a dog and cat isolate of *Dipylidium caninum* from the United States using the Illumina platform at mean coverage depths of 45× and 26× and conducted comparative analyses with the reference draft genome. Complete mitochondrial genomes were used to confirm the genotypes of the isolates. Genomes of *D. caninum* canine and feline genotypes generated in this study, had an average identity of 98% and 89%, respectively, when compared to the reference genome. SNPs were 20 times higher in the feline isolate. Comparison and species delimitation using universally conserved orthologs and protein-coding mitochondrial genes revealed that the canine and feline isolates are different species. Data from this study build a base for future integrative taxonomy. Further genomic studies from geographically diverse populations are necessary to understand implications for taxonomy, epidemiology, veterinary clinical medicine, and anthelmintic resistance.

## 1. Introduction

*Dipylidium caninum* (Linnaeus, 1758) is a cosmopolitan cestode belonging to the family Dipylidiidae of the order Cyclophyllidea. It is capable of infecting domestic dogs, domestic cats [1], wild carnivores [2], and humans [3]. Taxonomically, it is currently accepted that *D. caninum* occurs as two distinct, host-associated genotypes: the “*D. caninum* canine genotype” and the “*D. caninum* feline genotype” [4,5].

Definitive hosts acquire infection through ingesting the cysticercoid stage present within intermediate insect hosts—adult fleas of the genus *Ctenocephalides*, *Pulex*, or adult lice of the genus *Felicola* [6,7,8,9]. Cysticercoids are released within the intestines and develop into a scolex, followed by the development and maturation of immature and later gravid proglottids. Gravid proglottids are released into the intestines and pass out in feces. Gravid proglottids released into feces may move around the perineal region or bedding/furniture. These may occasionally cause pruritis of the peri-anal region resulting in scooting behavior in dogs. Proglottid disintegration or active extrusion releases egg packets containing 5–30 oncospheres, allowing for ingestion of the oncospheres by the larvae/juveniles of the intermediate hosts. Thus, the life cycle is indirect, and *D. caninum* infection in dogs and cats is often associated with an infestation of fleas or lice.

*D. caninum* has a moderately broad host-specificity. Two distinct, host-associated genotypes in dogs and cats is known. These genotypes were first demonstrated in phylogenetic analyses of partial 28S genes [8], partial mitochondrial 12S genes [9], and complete mitochondrial genomes [5]. In most naturally infected cases, dog and cat hosts are infected with their respective genotypes. Cat lice-derived and flea-derived *D. caninum* from Malaysia belonged to the feline genotype based on 12S analyses [9]. Seven of nine cat-derived *D. caninum* isolates from the United States belonged to the feline genotype at the 28S gene (genotypes from two isolates could not be determined) [5]. Dog flea-derived *D. caninum* from across Europe belonged to the dog genotype (100%), whereas 95.1% of cat flea-derived *D. caninum* from the same area belonged to the feline genotype based on 28S analyses [5]. Praziquantel resistant *D. caninum* samples from the United States obtained from dog feces were found in the canine genotype clade at both the partial 12S and 28S genes, showing host association [10].

This genotype distinction was further substantiated by in vivo experimental studies. Prepatent periods of the infection were shorter, and lifespans longer when host-associated genotypes infected the appropriate host [4]. There was no evidence of in vivo hybridization between the feline and canine genotypes. Host specificity and preference broke down in only 2–10% of natural infections [5]. In these cases, the feline genotype c be recorded in dogs or fleas isolated from dogs and vice versa. Experimentally, dogs are “permissive” to infection by the feline genotype, and cats are “permissive” to infection by the canine genotype [4]. Wildlife—hyaenas and red foxes—appear permissive to infections with the feline genotype [2,11]. Humans are also permissive to infection by the feline genotype [12]. Despite the permissivity, genotypes show biological adaptation with improved longevity and shorter prepatent periods in their respective hosts.

To date, no comparisons of canine and feline genotypes have been made at the whole genome level. If the two genotypes are two species, clinical implications exist for veterinarians who treat and control infections in the face of an increase in anecdotal reports of praziquantel resistance. 

Genetic differences at specific nuclear and/or mitochondrial genes and at the genome level are useful for differentiating species through species delimitation algorithms. Species delimitation has been used to resolve taxonomic conundrums in cestodes such as *Mesocestoides* [13] and other eukaryotes [14]. Recently, universal single-copy orthologs (USCOs) have been demonstrated to provide high resolution to differentiate between closely related species [15]. USCO genes from cat isolates of *D. caninum* have not been described yet. USCO genes from a dog isolate of *D. caninum* from China are available along with a draft nuclear genome [16], which can serve as a reference in comparative studies.

Our objectives in this study were to sequence the genomes of *D. caninum* isolated from a dog and a cat from the United States using the Illumina platform and to compare them to the reference *D. caninum* genome isolated from a dog in China [16]. This is the first study to sequence the whole genome of a feline isolate of *D. caninum* and perform comparative analyses of the genomes and SNPs of host-associated genotypes to improve our understanding of *D. caninum* biology with implications for treatment and control of animal infections. We hypothesized that the genomes of *D. caninum* canine isolates would be similar despite the geographical distance between the sites of isolation and that *D. caninum* feline isolates would have significant differences. Mitochondrial genomes were used to confirm the identity of the dog and cat isolates. Genomes were compared, and a set of single-copy orthologues were used in phylogenetic and species delimitation analyses.

## 2. Materials and Methods

### 2.1. Parasite Material and Sequencing

Feces of a dog in Florida, USA, naturally infected with *D. caninum*, were collected (isolate: Canine FL1). Proglottids were isolated by mixing the feces with water and sieving through a 1 mm sieve. Proglottids were removed from the sieve using forceps, thoroughly washed with 1x phosphate-buffered saline, and identified using egg packet and proglottid morphology. *D. caninum* proglottids passed by a cat in Kansas were isolated from the perineal area, washed thoroughly in 1x phosphate-buffered saline, and identified by morphology of egg packets and proglottids (isolate: Feline KS1). All proglottids were stored in 70% ethanol at −20 °C until DNA extraction.

Genomic DNA was extracted using the DNeasy Blood and Tissue Kit (Qiagen, Valencia, CA, USA) according to the manufacturer’s protocol. An RNase treatment was performed to remove co-purified RNA. Sample quantity was assessed using a Qubit fluorometer (Thermo Fisher Scientific, Waltham, MA, USA). Sample quality was assessed using an Agilent 5400 Bioanalyzer (Agilent Technologies Inc., Santa Clara, CA, USA). Genomic DNA was first fragmented using Covaris in the 350 bp mode. Library preparation was performed with NEB Next Ultra DNA Library Prep Kit (New England BioLabs Inc., Ipswich, MA, USA) following manufacturer’s instructions. Briefly, blunt ends fragments were generated by end-repairing 3′ or 5′ overhangs of double-stranded DNA (dsDNA) fragments followed by 3′ dA-tailing, index adapter ligation and size selection using SPRIselect beads. This was followed by PCR enrichment of the adaptor-ligated library. Samples were pooled and sequenced on Illumina HiSeq 4000 sequencer for 150 bp read length in paired-end mode, with an output of 17.9 million paired-end reads for the cat sample and 18.9 million paired-end reads for the dog sample. Raw data are publicly available on NCBI BioProject Accession: PRJNA768484; Sequence Read Archive (SRA) Accessions: SRX12485835, SRX12485836.

### 2.2. Assembly, Mapping, and Variant Analysis

FastQC (version 0.11.9) [17] was used to assess the sequence quality before and after adapter trimming with Trimmomatic (version 0.36) [18]. Mitochondrial genomes were assembled using Novoplasty (Version 4.3.1) [19], annotated with MITOS2 [20], and manually curated. Mitochondrial genomes were submitted to GenBank (Accession numbers: OK523384.1, OK523385.1). Identity of the genotypes was confirmed by BLAST [21] comparisons to previously described mitochondrial genomes [5,22,23]. Complete mitochondrial genomes generated from the study confirmed host-associated genotype identity.

Raw reads from the *D. caninum* Canine FL1 and Feline KS1 isolates were mapped to the previously described draft reference genome (Assembly Accession number: GCA_017562135.1) using BWA-MEM2 (Version 2.2.1) [24]. Coverage of the genomes was assessed using Qualimap (Version 2.2.2) [25] and bamCoverage (Version 3.5.1) [26] and then visualized with IGV-Web [27]. Variant analysis was conducted with DeepVariant [28]. Reference-guided assembly of draft genomes of the two isolates from this study was created with bcftools [29]. De novo assemblies were created with SPAdes (Version 3.15.4) [30]. Similarities and one-to-one comparisons between the genomes were conducted using dnadiff [31]. Assembly, mapping, and variant analysis were conducted on Galaxy servers [32].

The number of variants at each scaffold of the reference genome was plotted using vcfR (Version 1.13.0) [33] and *ggplot2* [34] in R. Summary statistics of the variant analysis were plotted with *ggplot2* [34] in R.

### 2.3. Benchmarking Universal Single-Copy Orthologs (BUSCO)

Genome completeness was assessed by Benchmarking Universal Single-Copy Orthologs (BUSCO) (Version 5.2.2) with metazoan lineage parameters in genome mode with the metaeuk predictor [35]. A total of 954 BUSCO groups were searched for each draft genome. Complete BUSCO genes were extracted from the draft assemblies from this study and the reference genome of *D. caninum* using bedtools (Version 2.30.0) [36] and parsed with biopython (Version 1.80) [37]. BUSCO completeness and genes present in the BUSCO sets between the three genomes were visualized with *ggplot2* and *ggvenn* in R (Version 4.1). For each gene, sequences were aligned with MAFFT (Version 7.487) [38] and trimmed with trimal (Version 1.2) [39]. Pairwise genetic distance matrices of the 503 complete BUSCO genes present in the three genomes were calculated using the TN93 model [40] with *apex* (Version 1.0.4) [41]. Heat maps of the calculated distances were created in *ComplexHeatmap* (Version 3.16) [42]. Principal component analysis of the SNPs present in the 503 BUSCO genes was analyzed with *adegenet* (Version 2.1.1) [43] and plotted with *ggplot2* [34].

### 2.4. Phylogenetic and Species Delimitation Analyses

The 3 sets of BUSCO genes obtained above and BUSCOs from 14 other cestode assemblies from GenBank were parsed with biopython (Version 1.80). Only BUSCO genes (128 genes) present in all 17 assemblies were used in the phylogenetic analysis. For each of 128 genes, sequences were aligned with MAFFT (Version 7.487) [38] and trimmed with trimal (Version 1.2) [39]. A concatenated supermatrix and gene partition file of the 128 BUSCO genes were created using *phylotools* (Version 0.2.2) [44] in R. Maximum likelihood phylogenetic reconstruction of the concatenated supermatrix was performed with IQtree2 (Version 2.1.0) [45] with ultrafast bootstrap approximation [46], using ModelFinder [47] to determine the best-fit model for each gene in the supermatrix (that is, partition model) [48], according to Akaike information criterion (AIC) scores and weights. The Diphyllobothridean clade represented by *Schistocephalus solidus* and *Spirometra erinaceieuropaei* was used as the outgroup. Species delimitation analyses of the trees were carried out using Bayesian PTP [49] and ASAP with the 2-parameter Kimura-80 model [50].GenBank records of complete mitochondrial genomes of cestodes of veterinary interest were obtained. Nucleotide sequences of mitochondrial protein-coding genes (12 genes) were parsed with the GenBank Feature Extractor [36]. A concatenated supermatrix and partition file of the 12 protein-coding genes were created in *phylotools* (Version 0.2.2) [44] in R. Maximum likelihood phylogenetic reconstruction of the concatenated supermatrix was performed with gene partitions as described for BUSCO genes. The mitochondrial genome of *Schistosoma mansoni* was used as the outgroup. Species delimitation analyses of the mitochondrial genome dataset were carried out as described for BUSCO genes.

## 3. Results

### 3.1. Identity Confirmed with Complete Mitochondrial Genomes

Proglottids were identified as *D. caninum* based on morphology. Complete mitochondrial genomes generated from the Illumina dataset were used to confirm host-associated genotype identity. The mitochondrial genomes from the *D. caninum* Canine FL1 and Feline KS1 genomes generated in this study were 14,296 bp and 13,598 bp long, respectively. The difference in length agrees with previously described mitochondrial genome lengths [5,22,23]. The complete mitochondrial genome of the *Dipylidium caninum* Canine FL1 isolate (Accession number: OK523384.1) had 97.65–99.82% identity with the genomes of canine isolates described earlier [22,23]. However, when the mitochondrial genome of *D. caninum* Canine FL1 was compared to the mitochondrial genomes of the feline isolates (This study and ref [5]), identity was only 84.25–86.21% (Table S1). The mitochondrial genome of the *Dipylidium caninum* Feline KS1 isolates from this study had 99.51% identity with the mitochondrial genome of the feline isolate previously described [5].

### 3.2. Quality Summary of the Datasets

**Figure 1:**
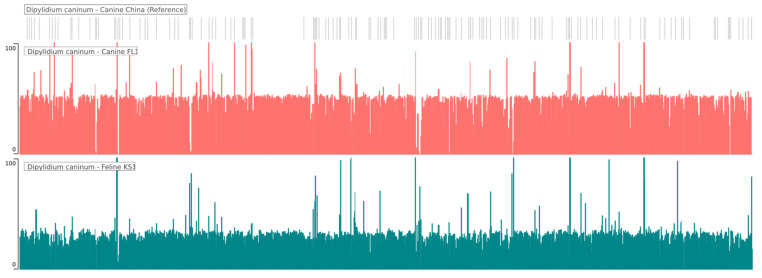
Whole genome coverage plot of *Dipylidium caninum* canine isolate FL1 (red-orange), and *Dipylidium caninum* feline isolate KS1 (teal) mapped to the reference *Dipylidium caninum* draft genome (grey). The average depth of coverage was 46.5× for the canine FL1 isolate and 25.8× for the feline KS1 isolate.

*D. caninum* Canine FL1 and Feline KS1 isolates from this study generated 18,886,666 and 17,928,712 raw reads (SRA Accessions: SRX12485835, SRX12485836), of which 18,827,832 (99.68%) and 17,870,800 (99.71%) reads were paired, with index trimmed mean insert lengths of 137.4 bp and 138.6 bp respectively. Trimmed reads were aligned with the *D. caninum* canine reference draft genome (Assembly Accession number: GCA_017562135.1) [16], which has 1686 scaffolds (Figure 1). The average depth of coverage of the *D. caninum* canine FL1 and feline KS1 genomes generated in this study were 46.5× and 25.8× with a GC% of 47.71% and 47.33%, respectively. Draft genomes generated using the reference guided assembler generated assemblies that were 108.97 Mb and 108.99 Mb for the canine and feline isolates, respectively; de novo assemblies were 120.43 Mb and 133.53 Mb, respectively.

### 3.3. Genomic Differences and Variation

**Figure 2:**
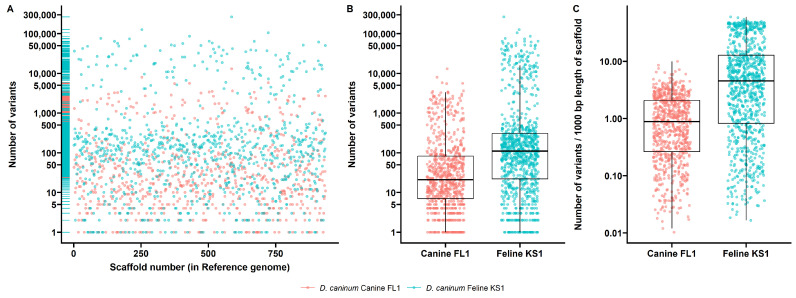
Genetic variants in the genomes *D. caninum* canine FL1 and *D. caninum* feline KS1 compared to the reference genome of *D. caninum.* (**A**) Scatterplot showing the number of genetic variants (SNPs, Indels) at each scaffold location. (**B**) Boxplots showing the distribution of the number of variants in each genome. (**C**) Boxplots showing the distribution of the mean number of variants per 1000 base pairs of reference scaffold.

**Figure 3:**
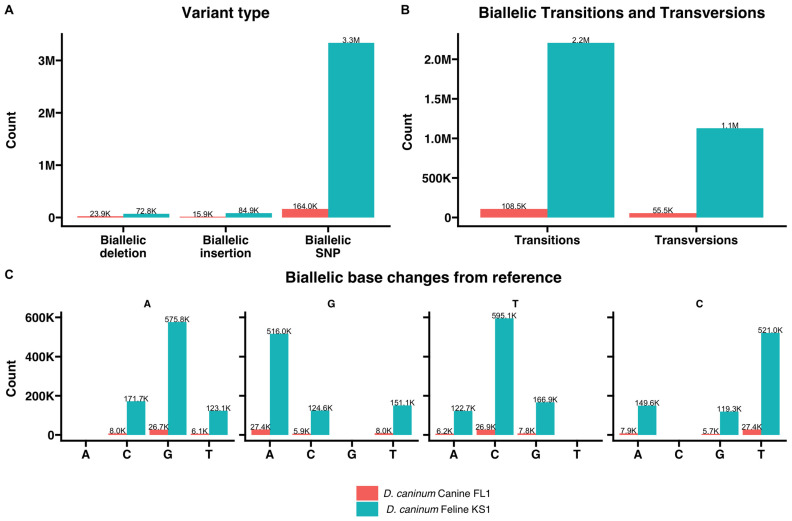
Summary of genetic variants in *D. caninum* canine FL1 and feline KS1 genomes from this study compared to the *D. caninum* canine reference genome. (**A**) Total counts of variants in the genomes by type is shown. (**B**) Counts of transitions and transversions in each genome are shown. (**C**) Counts of specific nucleotide base changes from reference for each compared genome are shown.

*D. caninum* Canine FL1 and Feline KS1 isolates from this study were compared to the scaffolds of the reference genome, and genetic variants were determined from mapped reads. The total number of variants that passed quality checks were 204,341 and 3,495,868 in the *D. caninum* Canine FL1 and Feline KS1 isolates, respectively. These variants were found across 741 and 905 scaffolds of the reference genome in the comparisons respectively. Mapping of variant counts across the scaffolds of the reference genome is shown in Figure 2A. Mean number of variants per scaffold was 275.8 (median 21) and 3863 (median 110) in the *D. caninum* Canine FL1 and Feline KS1 isolates, respectively (Figure 2B). To account for the length of each reference scaffold, mean number of variants per 1000 base pairs of reference scaffold was 1.31 (median 0.88) and 10.98 (median 4.54) in the *D. caninum* Canine FL1 and Feline KS1 isolates, respectively (Figure 2C). There were 3.3 million biallelic SNPs in the Feline KS1 isolate when compared to the reference genome, which was higher than the 164,000 SNPs in the Canine FL1 isolate (Figure 3A). Biallelic insertions and deletions were also higher in the Feline KS1 isolate than in the Canine FL1 isolate. Transition/transversion ratio was 1.95 and 1.96 in the Canine FL1 and Feline KS1 isolates, respectively (Figure 3B). Biallelic base changes from reference are shown (Figure 3C).

The draft genome sequences of the *D. caninum* Canine FL1 and Feline KS1 isolates from this study were compared for identity with each other and the reference genome. The Canine FL1 isolate had an average identity of 99.01% when compared to the reference genome in one-to-one alignments, whereas the Feline KS1 isolate had an average identity of 88.89% when compared to the reference genome. The draft genomes of the *D. caninum* Canine FL1 and Feline KS1 isolates from this study were 88.98% identical. Thus, a ~11% sequence identity difference exists between the genomes of *D. caninum* canine and feline genotypes.

### 3.4. BUSCO Statistics and Comparisons

**Figure 4:**
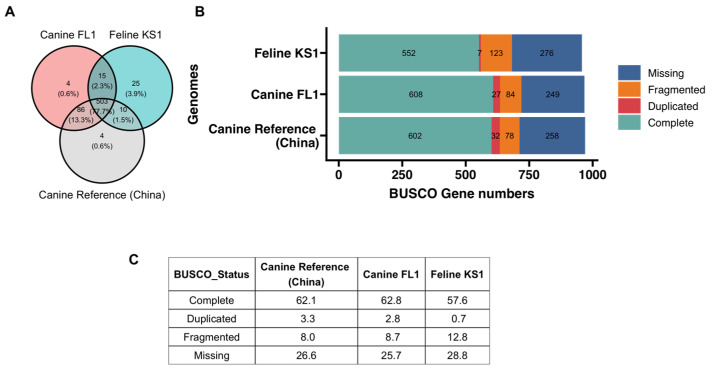
(**A**) Venn diagram of overlapping complete BUSCO genes among the three genomes. (**B**) BUSCO assessment results of the three genomes of *D. caninum.* (**C**) Percentages of BUSCO genes.

Complete and single copy BUSCO genes from the Canine FL1 and Feline KS1 isolates were 608 (63.7%) and 552 (57.9%) in number, respectively, out of the 954 tested in the metazoan lineage. BUSCO genes in the reference assembly (Assembly Accession number: GCA_017562135.1) were 602 (63.1%) (Figure 4). There were 503 orthologs present in all three assemblies. Nucleotide sequences of BUSCO genes of the two isolates from this study were compared with each other and the reference genome. BUSCOs from the Canine FL1 isolate had an average identity of 99.43% when compared to BUSCOs from the reference genome in one-to-one alignments, whereas BUSCOs from the Feline KS1 isolate had an average identity of 91.36% when compared to the BUSCOs from the reference genome. BUSCO genes from the Canine FL1 and Feline KS1 isolates from this study were 91.53% identical.

**Figure 5:**
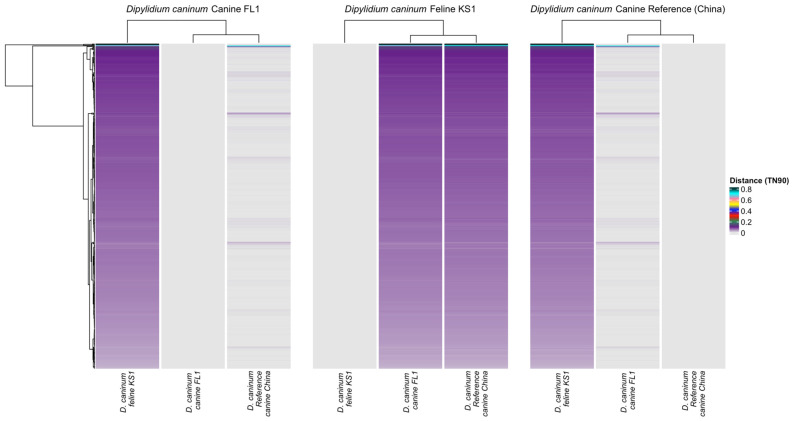
Heatmap of distance matrices at 503 complete BUSCO loci calculated with the Tamura-Nei 1993 model. Each line in the heatmap represents one gene. Color legend indicates genetic distance. A. BUSCO genes of *D. caninum* canine FL1 compared to *D. caninum* feline KS1 and *D. caninum* Reference canine China. B. BUSCO genes of *D. caninum* feline KS1 compared to *D. caninum* canine FL1 and *D. caninum* Reference canine China. C. BUSCO genes of *D. caninum* Reference canine China compared to *D. caninum* feline KS1 and *D. caninum* canine FL1.

Pairwise genetic distances were calculated at the BUSCO genes (503 genes) that were present in all three assemblies and mapped using a heatmap. Genetic distances between the genes of the two compared canine isolates were closer than the distances between the genes of the feline isolate and the canine isolates (Figure 5).

**Figure 6:**
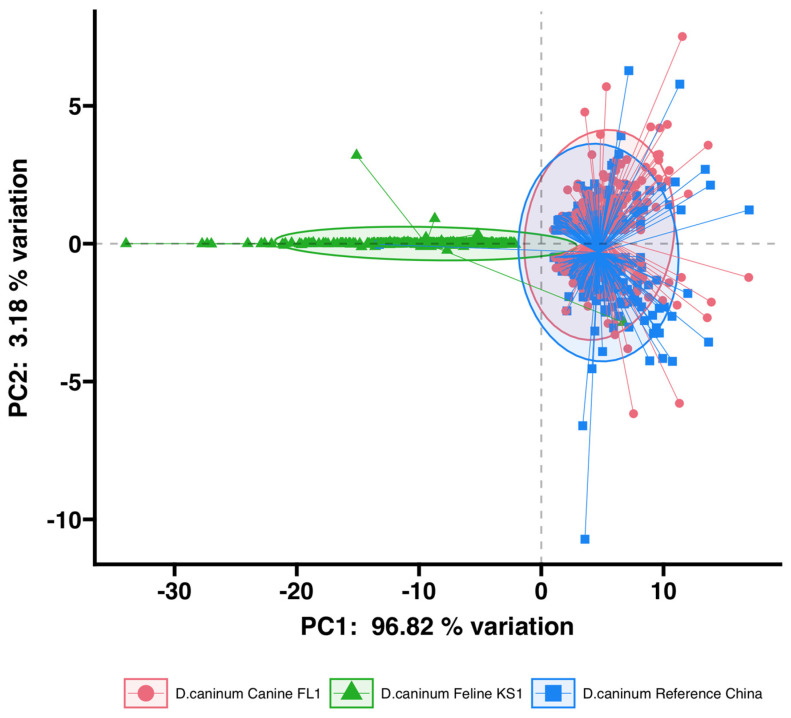
Principal component analysis plot of SNPs in the 503 BUSCO genes. 95% confidence intervals are shown as ellipses.

Additionally, a principal component analysis was performed to study the relationships between BUSCO genes from the three genomes. SNPs in the 503 shared BUSCO genes were used in the analysis. The first principal component explained 96.82% of the variation, and the second principal component explained 3.18% of the variation (Figure 6). The variances between the two canine isolates were similar, with overlapping 95% confidence intervals. There was little overlap in the 95% confidence intervals of the feline isolate and both the canine isolates.

### 3.5. Phylogenetic and Species Delimitation Analysis

**Figure 7:**
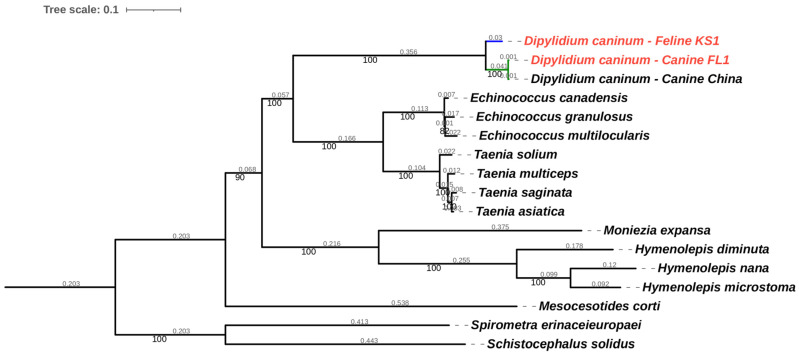
Maximum likelihood phylogenetic trees of 128 BUSCO genes of *Dipylidium caninum* genomes from this study and cestode genomes derived from GenBank, constructed using IQ Tree with gene-specific partition models. Accession numbers for genomes derived from GenBank and used in the BUSCO analysis are available in Table S2. Genomes from this study are highlighted in red. Species delimitation of the genus *Dipylidium* is highlighted.

**Figure 8:**
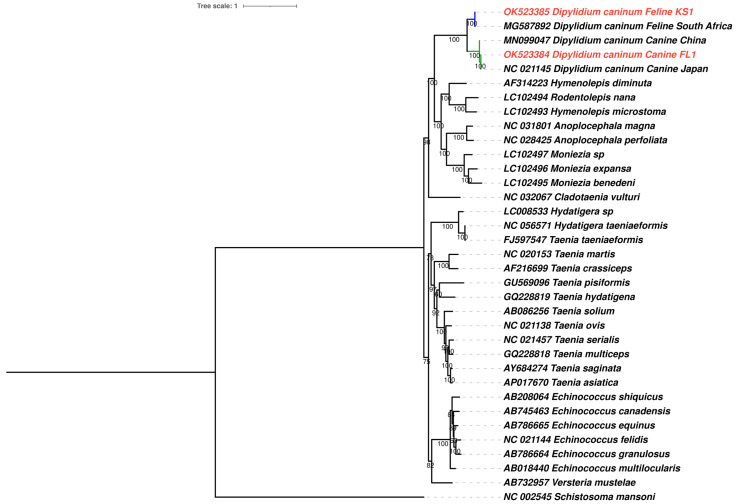
Maximum likelihood nucleotide phylogenetic tree of 12 mitochondrial protein-coding genes of *Dipylidium caninum* mitochondrial genomes from this study and those derived from GenBank, constructed using IQ Tree with gene-specific partition models. Each leaf of the tree has the GenBank accession and cestode species name. Mitochondrial genomes from this study are highlighted in red. Species delimitation of the genus *Dipylidium* is highlighted. A tree with branch lengths is available in Figure S8.

To understand phylogenetic relationships, a dataset of BUSCO genes from cestode genomes available in GenBank and those from this study was created. Full-length, complete genes that were present in all 17 genomes numbered 128 genes. These complete BUSCO genes were concatenated to form sequences of 297,029 nucleotide positions with gene partitions for maximum likelihood phylogenetic analysis, with gene-appropriate models for each gene. The analysis determined 168,880 sites to be parsimony-informative for the maximum likelihood analysis and 75,481 sites to be invariant. The canine isolate of *Dipylidium caninum* in this study formed a monophyletic clade with the canine isolate from China with high statistical support (100%), while the feline isolate KS1 formed a distinct branch (Figure 7). Species delimitation analysis of the BUSCO dataset and tree was carried out. Both the PTP Bayesian solution and ASAP ascending hierarchical clustering solution (Figure 8 colored branches, Figures S3 and S4) identified *D. caninum* Feline KS1 as a species distinct from the monophyletic clade of the two *D. caninum* canine isolates. The clade with the *D. caninum* canine isolates is supported as a distinct species. Interestingly, distances between the feline isolate, and the canine isolates were larger than the interspecies distances within the genera *Taenia* and *Echinococcus* (Figures S3 and S4)

Additionally, a maximum likelihood phylogenetic tree was constructed from a nucleotide mitochondrial genome dataset, created with the 12 protein-coding genes present in the mitochondrial genomes from this study and other cestode mitochondrial genomes available in GenBank (Figure 8). Protein-coding genes were concatenated to form a supermatrix of 9948 nucleotide positions with gene partitions for maximum likelihood phylogenetic analysis, with gene-appropriate models for each gene in the supermatrix. The analysis determined 5959 sites to be parsimony-informative for the maximum likelihood analysis and 2817 sites to be invariant. While the genus *Dipylidium* is monophyletic, there are two distinct clades within it, formed by feline and canine isolates of *D. caninum.* Mitochondrial genomes of *D. caninum* from this study were located within their host-associated clades. Both clades have high statistical support (100%). Bayesian solution-based species delimitation provided support for the two clades to be considered distinct species (Figure 7 colored branches, Figure S5). Hierarchical clustering-based species delimitation provided two solutions when the prior known species distinctions were applied (Figure S7). The first solution provides evidence for distinct species designations to the host-associated clades, with the feline and canine isolates from this study and prior studies belonging to two distinct species. The second solution suggests that the mitochondrial genome (Accession number: MN099047.1) described from China [22] is a distinct species within the *D. caninum* canine clade. MN099047 could represent a cryptic species. However, species delimitation of the feline isolates was present even when the second solution was accepted.

## 4. Discussion

*Dipylidium caninum* is a zoonotic cestode that belongs to the family Dipylidiidae within the order Cyclophyllidea. In this study, we show for the first time that the canine and feline genotypes of *D. caninum* are distinct in the nuclear genome. In whole genome comparisons, an 11% difference was found to exist between the two genotypes. In representative universal single-copy ortholog gene comparisons (503 genes), 8.47–8.64% differences were calculated between the two genotypes. In complete mitochondrial genome comparisons, 13.79–15.83% differences were calculated between the two genotypes. Applying species delimitation criteria to the nuclear and mitochondrial genome data suggest that the canine and feline genotypes represent two species. We discuss these findings in the context of genomic differences, host specificity, and clinical applications.

Variants were found across the genome in both the Canine FL1 and Feline KS1 isolates from this study, with higher numbers of variations in the Feline KS1 genome in comparative analyses with the reference genome described by Liu et al. [16] (Figure 2). SNPs and InDels found in the genomes may cause significant protein changes in the two genotypes. They may also be of diagnostic significance in addition to the partial 28S rDNA gene to differentiate the two genotypes. Further studies with several host-associated isolates are essential to understand SNPs and InDels that are present in all feline and all canine genotype isolates.

Based on pairwise genetic distances, BUSCO genes in canine genotypes (*D. caninum* Canine FL1 and *D. caninum* reference genome) are more similar to each other than BUSCO genes between the feline genotype (*D. caninum* Feline KS1) and the canine genotypes (Figure 5). Two distinct clusters with little overlap—the feline genotype cluster and the canine genotype cluster—were found in the principal component analysis (Figure 6). In phylogenetic and species delimitation analyses of more than 500 BUSCO genes (Figure 7), the canine and feline genotypes were distinct species. Additionally, phylogenetic and species delimitation analyses of protein-coding mitochondrial genes (Figure 8) provide evidence for the designation of distinct species identities to the canine and feline genotypes.

Nuclear-mitochondrial discordance is evident in the position of the monophyletic clade *D. caninum* in relation to other cestode genera. In the nuclear BUSCO gene phylogeny, the family Dipylidiidae (represented by *D. caninum*) was more closely related to the family Taeniidae (represented by *Echinococcus* spp. and *Taenia* spp.) than to the families Anoplocephalidae (represented by *Moniezia expansa*) and Hymenolepididae (represented by *Hymenolepis* spp.) However, in the mitochondrial gene phylogeny, family Dipylidiidae was more closely related to the families Anoplocephalidae (represented by *Moniezia* spp. and *Anoplocephala* spp.) and Hymenolepididae (represented by *Hymenolepis* spp.) than to the family Taeniidae (represented by *Taenia* spp., *Hydatigera* spp., *Echinococcus* spp., and *Versteria* sp.).

Currently, *D. caninum* is the only valid species within the genus *Dipylidium* [51]. It is current practice to morphologically identify any armed medium-sized cestodes isolated from dogs and cats with a retractable rostellum, double-pored proglottids, and eggs present within egg capsules as *D. caninum.* The two genotypes of *D. caninum* appear to be morphologically indistinguishable, and a wide range of variations is considered normal within the species [52]. Despite the lack of marked morphological features, a reassessment of the taxonomy of the genus *Dipylidium* based on recent biological and molecular research from the last decade is due. The present study is the first to provide additional evidence using whole genome data to the splitting of *D. caninum* into two species as proposed by Labuschagne et al. [5].

The designation of species status to the canine and feline genotypes has clinical implications for veterinarians in small animal practices and in shelter situations. Based on previous work, the likelihood of encountering host-associated *Dipylidium* spp. in pet dogs and cats is higher than the likelihood of encountering non-host-associated species (2–10%) [5,8]. Dogs and cats in sympatry in multi-pet households and in shelters may share fleas and *Dipylidium* spp. cysticercoids within. Risk of *Dipylidium* spp. infection is higher when pets are flea infested. Further epidemiological work is essential to understand the prevalence of *Dipylidium* spp. cysticercoids in flea populations in different parts of the world and the vectorial capacity of different flea species since the relative abundance of flea species on pets varies across the world [53,54]. At the same time, praziquantel and epsiprantel are useful for treating *Dipylidium* spp. infections in both cats and dogs, veterinarians should be aware that praziquantel resistance has only been reported in canine isolates so far [10].

The small sample size used in this study is a potential pitfall. Although we compared the feline and canine genotypes for the first time, further genetic studies with geographically diverse isolates are essential to increase confidence in the genetic variant calls recorded in this study. The relatively low depth of coverage of the isolates is another drawback in the face of the increasing availability of sequencing techniques that provide high coverage [55]. While coverages of 20–30× are common in genomic studies [56], low coverage depths of 4–5× are now being used to detect known and novel variations in larger, more complex eukaryotic genomes [57]. Thus, despite the weaknesses, this study is expected to close a knowledge gap about the difference between the host-associated genotypes of *D. caninum* and can provide a base for integrative taxonomy studies in the future. In light of the new knowledge uncovered in this study, a taxonomic revision of the genus *Dipylidium* may be warranted.

## 5. Conclusions

In conclusion, we performed comparative analyses on the nuclear and mitochondrial genomes of dog and cat isolates of *Dipylidium caninum*, representing the canine and feline genotypes. Based on variations, genetic distances, phylogeny, and species delimitation from this study, in addition to biological differences previously demonstrated in experimental studies, there is adequate support for the canine and feline genotypes of *D. caninum* to belong to different species. A taxonomic revision of the genus *Dipylidium* is necessary.

## Data Availability

All data from this project is publicly available NCBI Bioproject Accession: PRJNA768484; Sequence Read Archive (SRA) Accessions: SRX12485835, SRX12485836. Mitochondrial genomes are available in GenBank (Accession numbers: OK523384.1, OK523385.1).

## Supplementary Materials

The following supporting information can be downloaded at: https://www.mdpi.com/article/10.3390/pathogens12050675/s1, Table S1. BLAST results of complete mitochondrial genomes; Table S2. Cestode species, GenBank accession numbers, and numbers of genes used in the BUSCO analysis. There were 128 ortholog genes present in all genomes listed; Figure S1. PCA showing the connected positions of components at each gene for each genome; Figure S2. Pairwise genetic distances (Tamura-Nei, 1990) of the 128 gene supermatrix of BUSCOs from cestode species; Figure S3. Output of bayesian PTP analysis of the ML phylogenetic tree created with a partitioned supermatrix 128 BUSCO genes in IQTree (See Figure 7). The tree was rooted on the Diphyllobothridean outgroup, with 100,000 MCMC generations, 100 thinning, and 0.1 burn-in; Figure S4. ASAP species delimitation output created with a fasta supermatrix of 128 BUSCO genes and Kimura 80 (Ti/Tv) substitution model; Figure S5. Pairwise genetic distances (Tamura-Nei, 1990) between concatenated 12 protein-coding mitochondrial gene datasets; Figure S6. Output of bayesian PTP analysis of the ML phylogenetic tree created with partitioned mitochondrial 12 protein-coding nucleotide supermatrix in IQTree (See Figure 8). The tree was rooted on Schistosoma mansoni, and the outgroup was removed to improve delimitation. Analysis was performed with 100,000 MCMC generations, 100 thinning, and 0.1 burn-in; Figure S7. ASAP species delimitation output created with a fasta supermatrix of mitochondrial 12 protein-coding genes and Kimura 80 (Ti/Tv) substitution model; Figure S8. Maximum likelihood nucleotide phylogenetic tree of 12 mitochondrial protein-coding genes of *Dipylidium caninum* mitochondrial genomes with branch lengths is available. References [5,22,23] are cited in the supplementary materials.
