# Supplementary material for: Comparative Genomic Analysis and Species Delimitation: A Case for Two Species in the Zoonotic Cestode Dipylidium caninum
Source: Pathogens. 2023 May 3;12(5):675. doi: 10.3390/pathogens12050675 (PMC10221840; doi:10.3390/pathogens12050675)
Supplement: Supplementary file 1 [file pathogens-12-00675-s001.zip › pathogens-2280124-supplementary.pdf]

Table S1. BLAST results of complete mitochondrial genomes

Accession<br>Length<br>Host<br>Reference	OK523384.1<br>14296 bp<br>Canine<br>(This study)	OK523385.1<br>13598 bp<br>Feline<br>(This study)	NC_021145.1<br>AB732959.1<br>14296 bp<br>Canine<br>(Nakao et al., 2013)	MN099047.1<br>14226 bp<br>Canine<br>(Xie et al., 2019)	MG587892.1<br>13598 bp<br>Feline<br>(Labuschagne et al., 2018)
**OK523384.1<br>14296 bp<br>Canine<br>(This study)**	100.00%	84.26%	99.82%	97.65%	84.17%
**OK523385.1<br>13598 bp<br>Feline<br>(This study)**	84.26%	100.00%	84.25%	86.21%	99.51%

Labuschagne, M., Beugnet, F., Rehbein, S., Guillot, J., Fourie, J., Crafford, D., 2018. Analysis of Dipylidium caninum tapeworms from dogs and cats, or their respective fleas - Part 1. Molecular characterization of Dipylidium caninum: genetic analysis supporting two distinct species adapted to dogs and cats. Parasite 25, 30.

Nakao, M., Lavikainen, A., Iwaki, T., Haukisalmi, V., Konyaev, S., Oku, Y., Okamoto, M., Ito, A., 2013. Molecular phylogeny of the genus Taenia (Cestoda: Taeniidae): proposals for the resurrection of Hydatigera Lamarck, 1816 and the creation of a new genus Versteria. Int J Parasitol 43, 427-437.

Xie, Y., Liu, Y., Gu, X., Meng, X., Wang, L., Li, Y., Zhou, X., Zheng, Y., Zuo, Z., Yang, G., 2019. Complete mitogenome of the dog cucumber tapeworm. Mitochondrial DNA B Resour 4, 2670-2672.

Table S2. Cestode species, GenBank accession numbers and numbers of genes used in the BUSCO analysis. There were 128 ortholog genes present in all genomes listed.

**Species**	**GenBank Accession for genomes**	**Number of complete and single-copy ortholog genes**
Hymenolepis microstoma	GCA_000469805.3	619
Hymenolepis nana	GCA_900617975.1	560
Hymenolepis diminuta	GCA_900708905.1	602
Spirometra erinaceieuropaei	GCA_902702965.1	501
Echinococcus multilocularis	GCA_000469725.3	511
Echinococcus granulosus	GCA_000524195.1	513
Taenia asiatica	GCA_001693035.2	490
Taenia saginata	GCA_001693075.2	501
Taenia solium	GCA_001870725.1	484
Taenia multiceps	GCA_001923025.3	509
Schistocephalus solidus	GCA_017591395.1	539
Moniezia expansa	GCA_019097775.1	531
Echinococcus canadensis	GCA_900004735.1	515
Mesocestoides corti	GCA_900604375.1	550
***Dipylidium caninum* (China)**	GCA_017562135.1	603

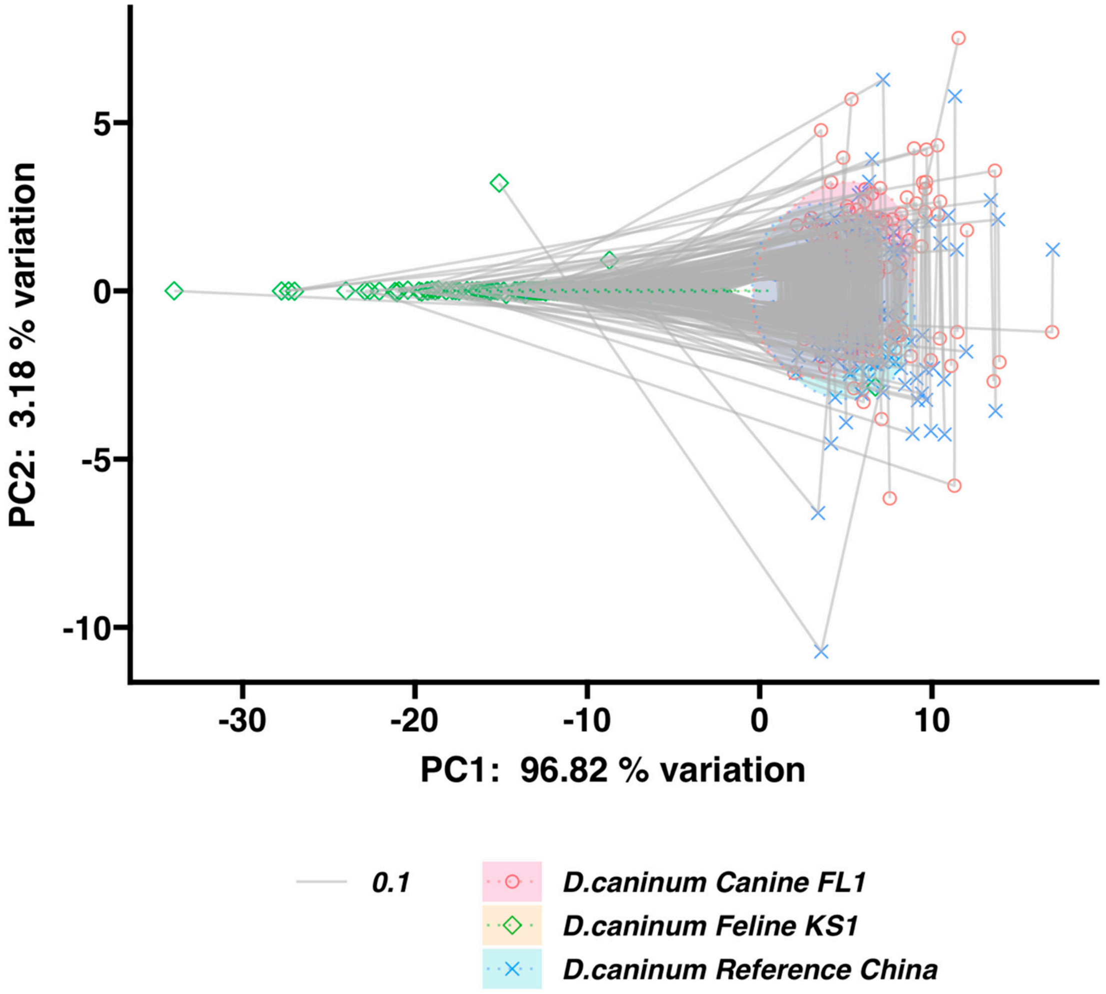

Figure S1. PCA showing the connected positions of components at each gene for each genome.

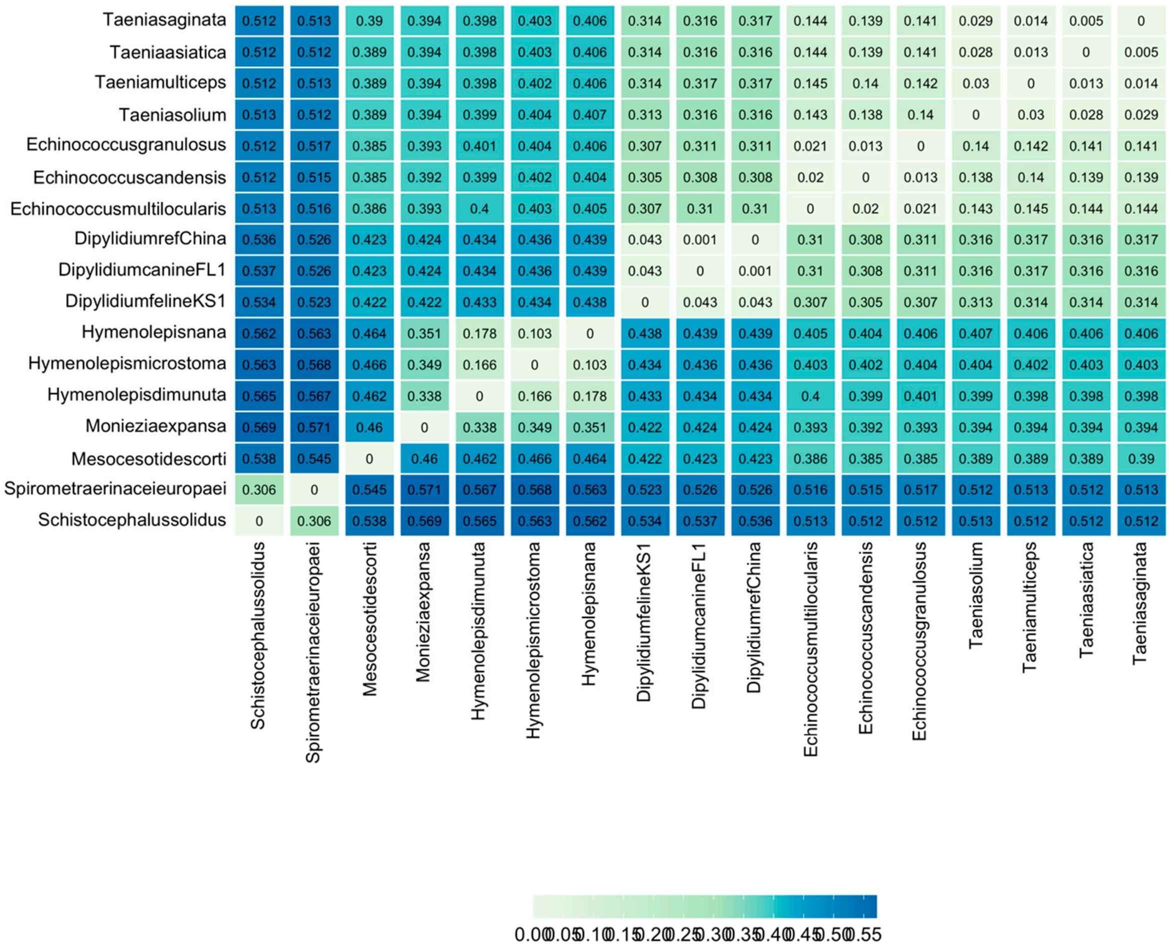

Figure S2. Pairwise genetic distances (Tamura-Nei, 1990) of the 128 gene supermatrix of BUSCOs from cestode species.

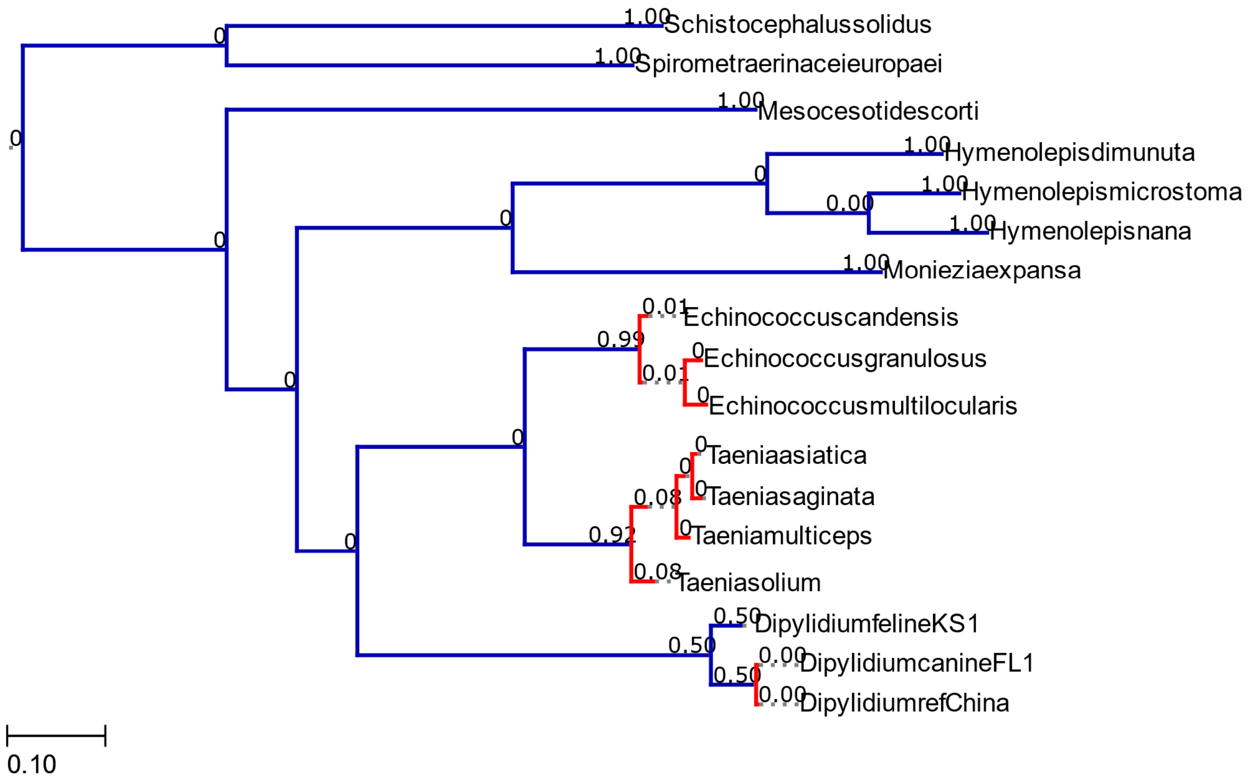

Figure S3. Output of bayesian PTP analysis of the ML phylogenetic tree created with a partitioned supermatrix 128 BUSCO genes in IQTree (See Figure 7). The tree was rooted on the Diphyllobothridean outgroup, with 100,000 MCMC generations, 100 thinning and 0.1 burn-in.

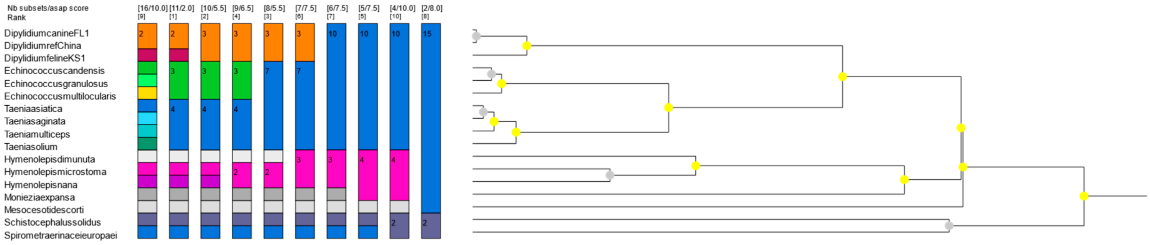

Figure S4: ASAP species delimitation output created with a fasta supermatrix of 128 BUSCO genes and Kimura 80 (Ti/Tv) substitution model.

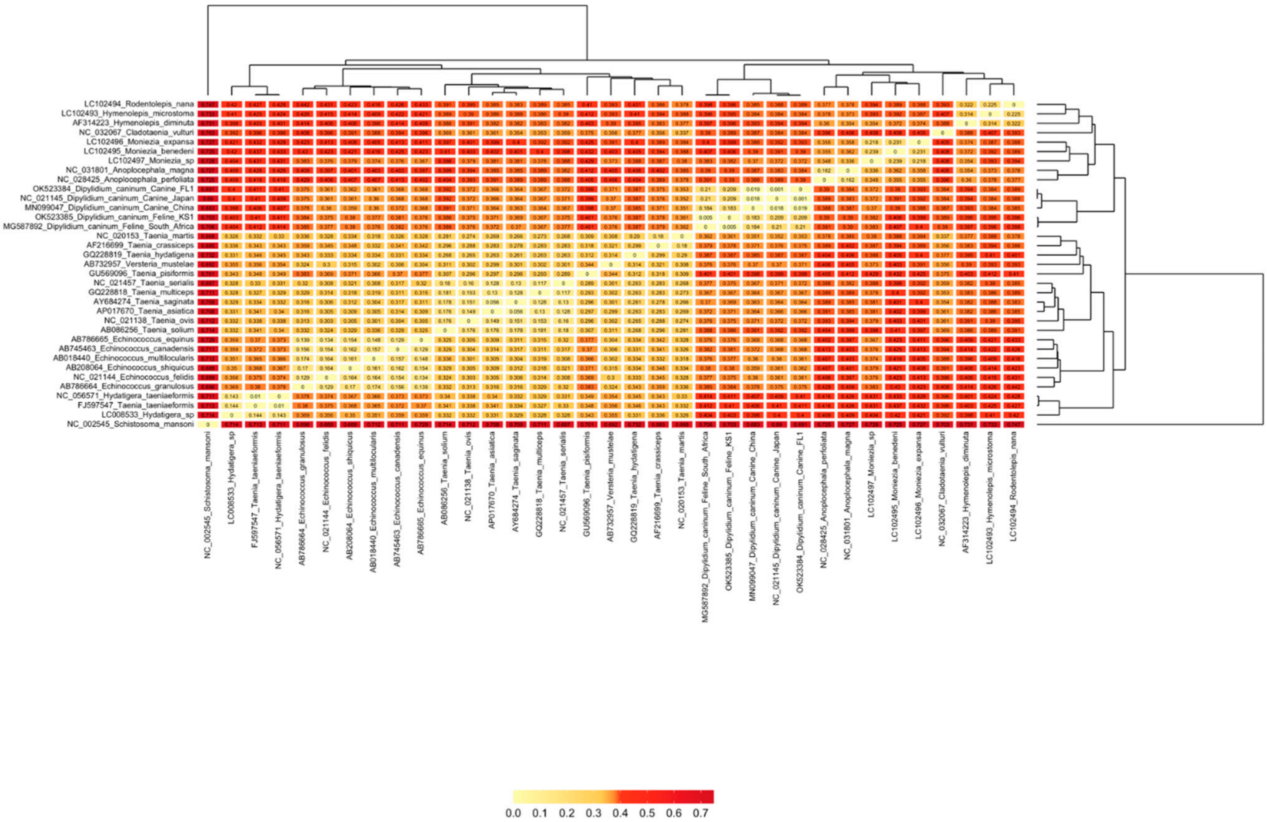

Figure S5. Pairwise genetic distances (Tamura-Nei, 1990) between concatenated 12 protein coding mitochondrial gene datasets.

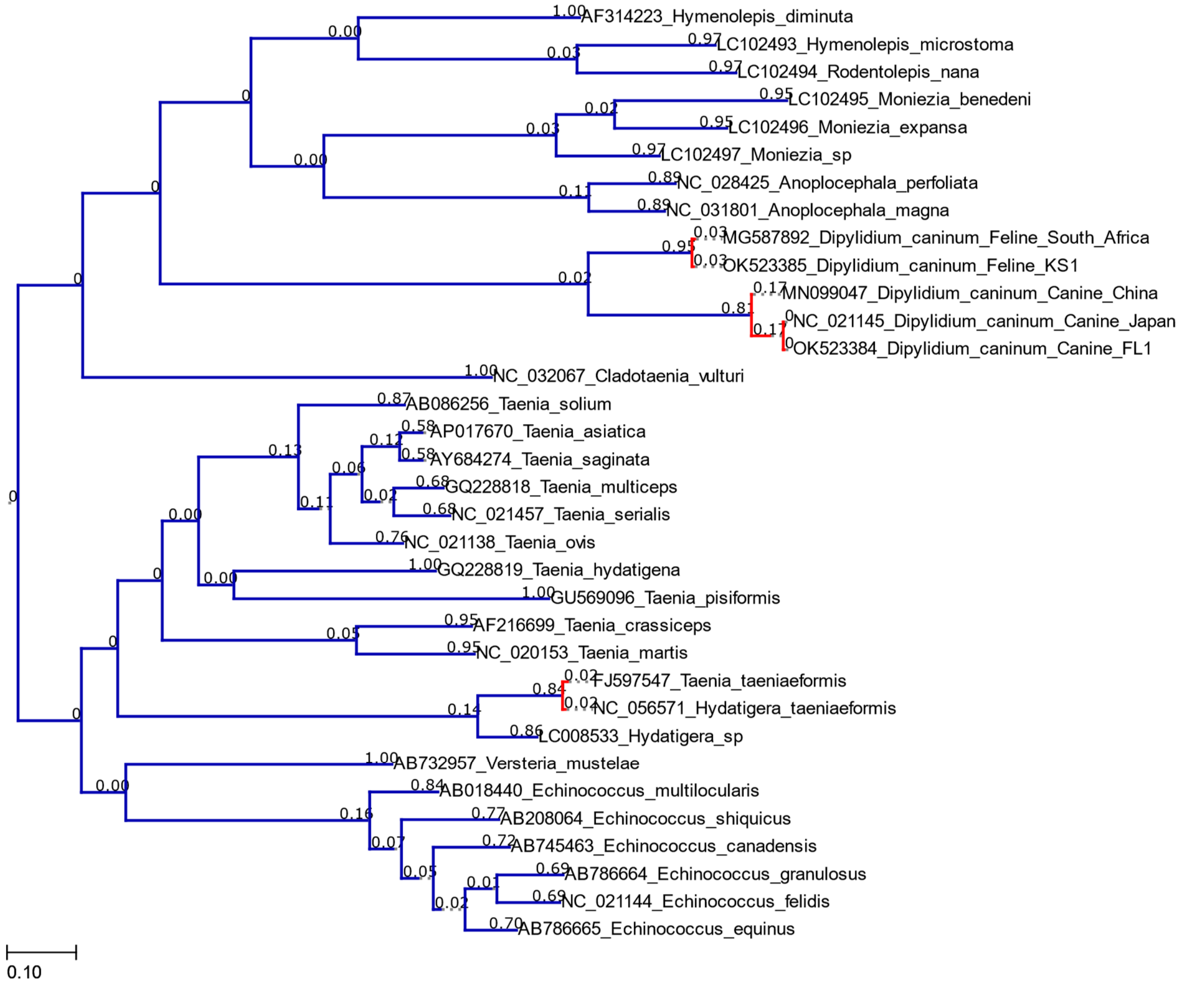

Figure S6. Output of bayesian PTP analysis of the ML phylogenetic tree created with partitioned mitochondrial 12 protein-coding nucleotide supermatrix in IQTree (See Figure 8). The tree was rooted on *Schistosoma mansoni* and the outgroup was removed to improve delimitation. Analysis was performed with 100,000 MCMC generations, 100 thinning and 0.1 burn-in.

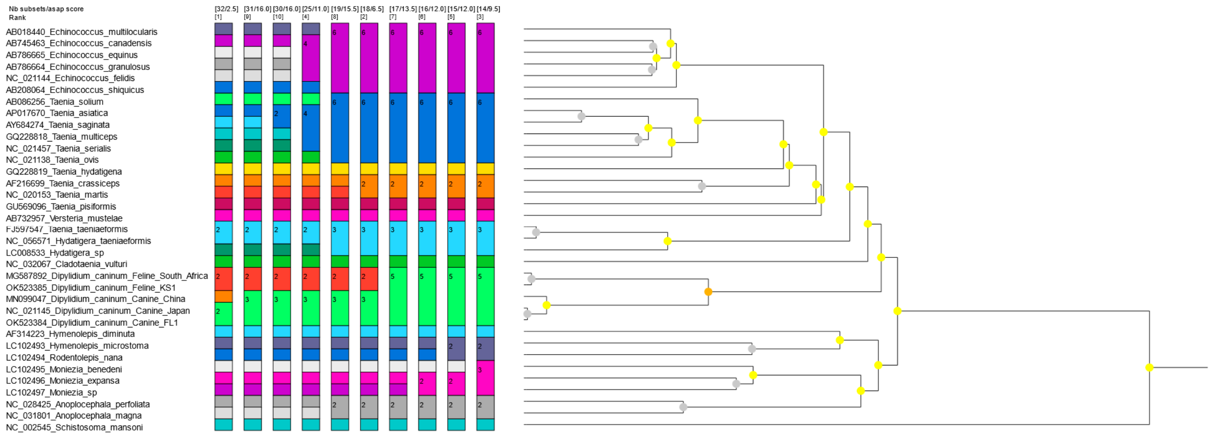

Figure S7: ASAP species delimitation output created with a fasta supermatrix of mitochondrial 12 protein-coding genes and Kimura 80 (Ti/Tv) substitution model.

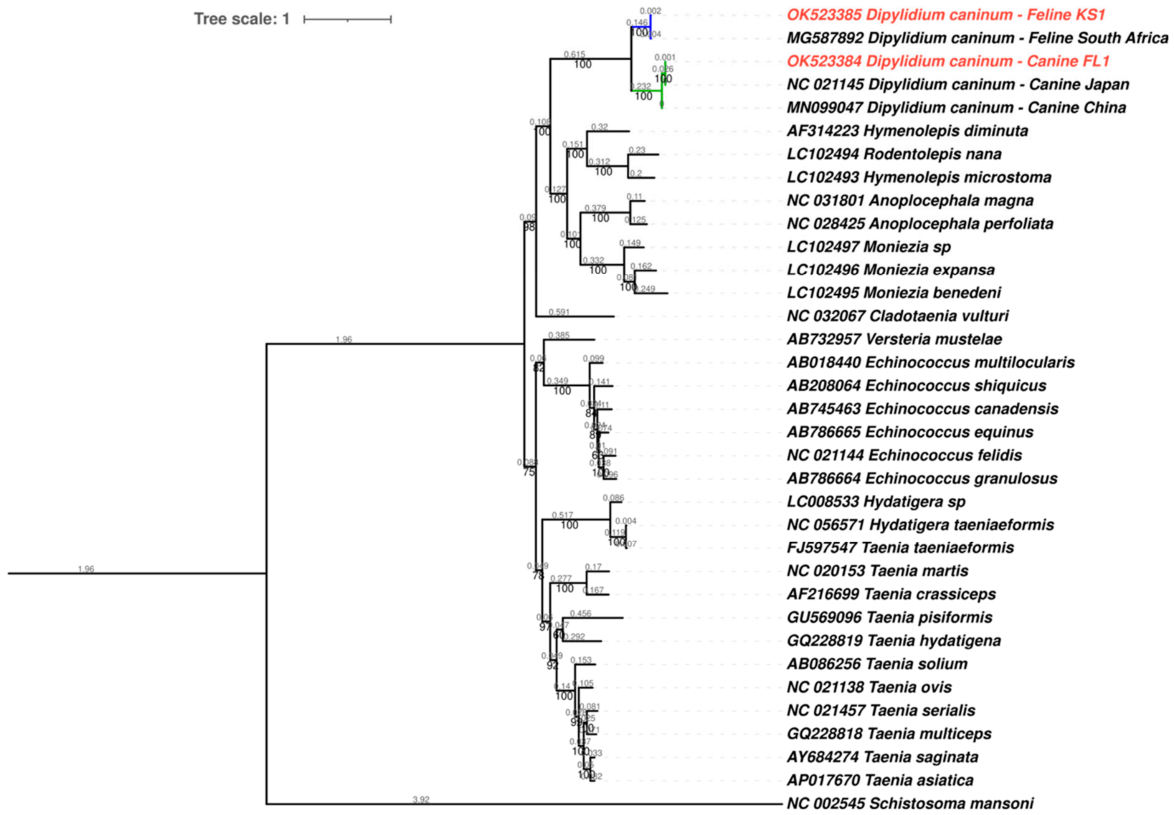

Figure S8. Maximum likelihood nucleotide phylogenetic tree of 12 mitochondrial protein coding genes of *Dipylidium caninum* mitochondrial genomes with branch lengths is available.
